# Target Localization and Tracking by Fusing Doppler Differentials from Cellular Emanations with a Multi-Spectral Video Tracker

**DOI:** 10.3390/s18113687

**Published:** 2018-10-30

**Authors:** Casey D. Demars, Michael C. Roggemann, Adam J. Webb, Timothy C. Havens

**Affiliations:** 1 Department of Electrical and Computer Engineering, Michigan Technological University, Houghton, MI 49931, USA; mroggema@mtu.edu (M.C.R.); ajwebb@mtu.edu (A.J.W.); thavens@mtu.edu (T.C.H.); 2 Michigan Tech Research Institute, Ann Arbor, MI 48105, USA; 3 Department of Computer Science, Michigan Technological University, Houghton, MI 49931, USA

**Keywords:** sensor fusion, target tracking, localization, identification

## Abstract

We present an algorithm for fusing data from a constellation of RF sensors detecting cellular emanations with the output of a multi-spectral video tracker to localize and track a target with a specific cell phone. The RF sensors measure the Doppler shift caused by the moving cellular emanation and then Doppler differentials between all sensor pairs are calculated. The multi-spectral video tracker uses a Gaussian mixture model to detect foreground targets and SIFT features to track targets through the video sequence. The data is fused by associating the Doppler differential from the RF sensors with the theoretical Doppler differential computed from the multi-spectral tracker output. The absolute difference and the root-mean-square difference are computed to associate the Doppler differentials from the two sensor systems. Performance of the algorithm was evaluated using synthetically generated datasets of an urban scene with multiple moving vehicles. The presented fusion algorithm correctly associates the cellular emanation with the corresponding video target for low measurement uncertainty and in the presence of favorable motion patterns. For nearly all objects the fusion algorithm has high confidence in associating the emanation with the correct multi-spectral target from the most probable background target.

## 1. Introduction

Detection and tracking of moving targets in cluttered urban environments is an important task for local law enforcement and security forces. The ability to locate and track a single target in a large cluttered scene is difficult due to several factors based on the target and its surrounding environment. For example, it is sometimes difficult to differentiate between targets when using a thermal infrared (IR) sensor as target signatures are similar and thermal contrast between the target and the background can be low. Radio frequency (RF) communications contain identification information about the transmitting source but lack the ability to spatially localize the target with low uncertainty [1]. The combination of data from two or more sensors, referred to as sensor fusion, exploits the advantages of multiple sensors while overcoming the disadvantages of each individual sensor [2,3].

RF transmissions have been instrumental for decades in detection and tracking of targets using both active and passive systems. Passive RF systems exploit existing sources of opportunity such as cellular communications or television broadcasts. These systems have been demonstrated in a number of applications such as surveillance [4,5], geolocation [6,7], and motion estimation [8]. A cellular phone is a device that emits an RF signal and has been of interest for surveillance by government agencies including local law enforcement and the federal bureau of investigation [9]. Cellular phones can be tracked by devices known as “stringrays” that act as a cellular tower and intercept the cellular signal to localize and track a specific target [10]. As cellular phones contain unique identifications, they are excellent sources to identify targets with high confidence.

Given their generally high angular resolution, and in the case of infrared sensing, night vision capability, electro-optical/infrared (EO/IR) sensors are commonly used to identify and track a variety of targets including pedestrians [11,12,13], vehicles [14], ships [15], and aircraft [16]. Under optimal viewing conditions EO/IR sensors can measure the location of a target with high accuracy and precision making them an important asset for security systems. Algorithms to accomplish these tasks have been demonstrated making use of background estimation [17,18,19], edge detection [20], and feature recognition [21]. That said, it is the case that EO/IR sensors cannot see inside most vehicles. Hence, in a crowded traffic environment where a particular cell phone is being used, associating the cell phone emanation with a particular vehicle is an important problem, which is addressed here.

A number of sensor combinations have been developed to aid in target detection and tracking applications. Noulas et al. fuse audio segments with a video sequence to associate the audio with its corresponding video target [22]. Kilic et al. track speaking targets by fusing likelihoods built from audio and visual data [23]. D’Arca et al. fuse audio and visual sensors to estimate a targets trajectory using separate Kalman filters that get fused into a single Kalman filter [24]. Chin et al. demonstrate a fusion technique using an optical tracker and Wi-Fi to track a target through obscurations [25]. We explore a fusion algorithm to localize and track a specific vehicle using two new sensor types; a constellation of RF sensors capturing cell phone emanations and a multi-spectral imaging system. To the best of our knowledge the fusion of these sensors is unique in the literature.

**Figure 1:**
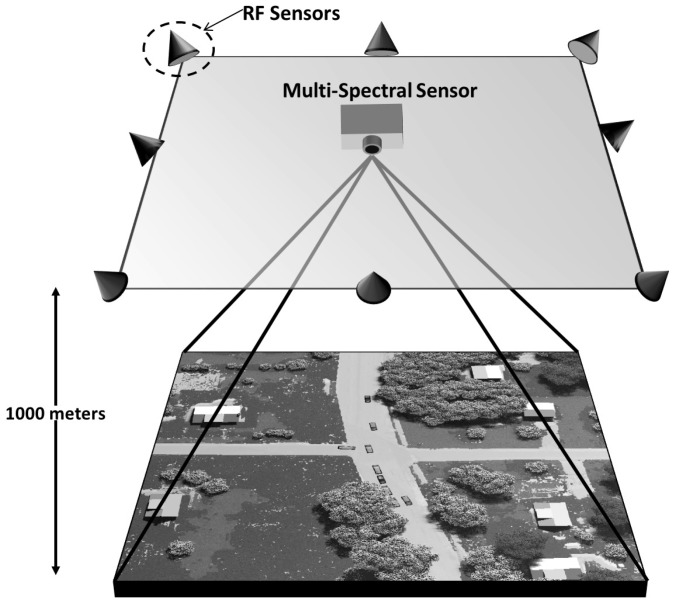
Geometry of the multi-spectral and RF sensors in reference to the plane in which the moving vehicles lie.

**Figure 2:**
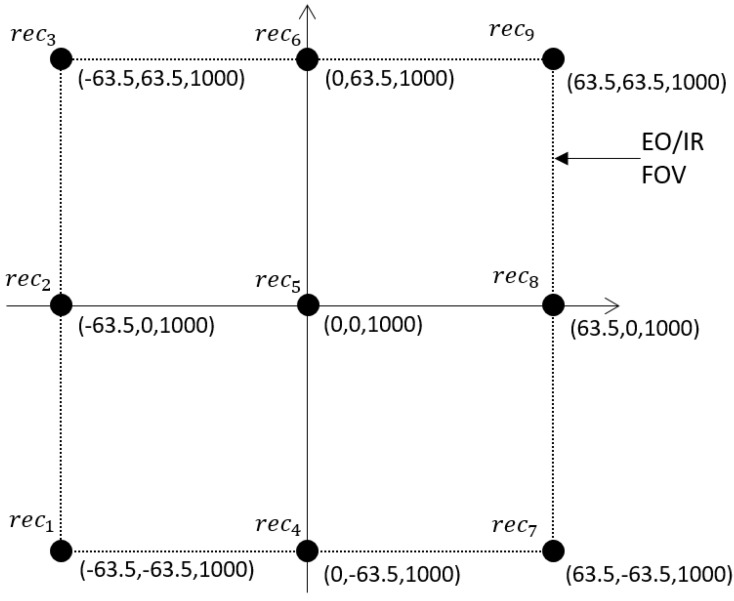
Coordinates of the multi-spectral and RF sensors in reference to the plane in which the moving vehicles lie.

We present a novel combination of passive sensor data fusion by using a constellation of RF sensors measuring a cellular emanation from a specific phone with a multi-spectral imaging sensor detecting and tracking vehicles in a target rich environment. In practice, neither signal contains enough information to allow a particular vehicle to be uniquely identified as the source of the cellular emanations. However, by fusing these two sources of data we demonstrate that a specific target can confidently be identified and tracked through a sequence of frames. From the cellular emanation we make use of the frequency difference of arrival (FDOA), also referred to as the Doppler differential (DD), which is a result of relative motion between the emitter and separated receivers [26,27]. The multi-spectral sensor produces centroid estimates of multiple moving vehicles through a sequence of frames. Constellations of unmanned aerial vehicles (UAV) have become readily available and demonstrated for various applications [28,29,30]; the notional sensor configuration studied here is a UAV scenario with a multi-spectral imaging sensor located at the scene origin at an altitude of 1000 m and RF sensors spaced on the border of the imaging sensors field of view at an altitude of 1000 m. The sensor-scene geometry is shown in Figure 1, and the sensor coordinates shown in Figure 2. The geometry of the RF sensors on the border of the scene is chosen to provide diversity to the DD of received signals; other geometries would work for this application.

**Figure 3:**
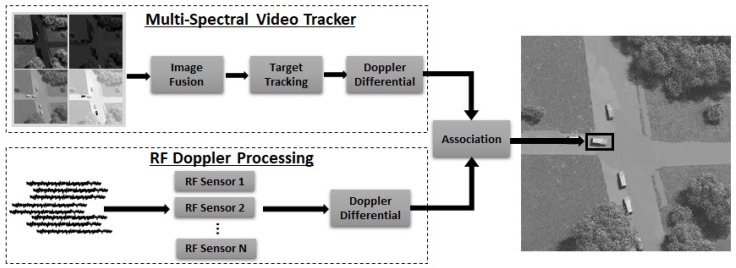
Flowchart of the fusion processing to associate a specific moving target between a multi-spectral video tracker and a constellation of RF sensors.

**Figure 4:**
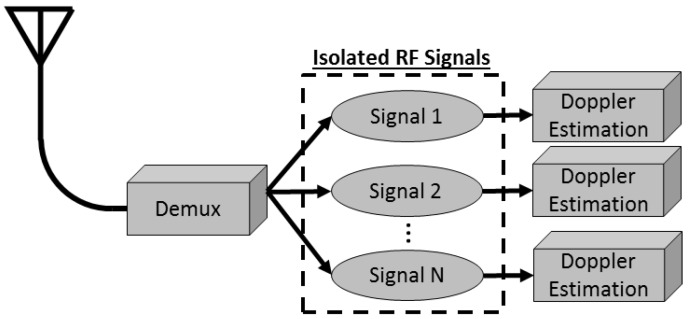
Flowchart of RF sensor processing to isolate and extract Doppler shifts.

The block diagram of the fusion algorithm described here is shown in Figure 3. The multi-spectral video tracker fuses images for detection of moving foreground objects which are then tracked through a video sequence [31], giving two dimensional time history of (x,y) centroid locations of multiple moving targets. Radial velocity estimates are computed from the tracker outputs and used to calculate the theoretical DD which would have been observed at the cell phone frequency for each tracked target. The RF receivers in the constellation each measure incoming cellular emanations and isolate a signal of interest and extract the Doppler shift. The RF process for an individual sensor is shown in Figure 4. It is not in the scope of this paper to cover the Doppler shift estimation, but we note this is a viable operation in wireless communications to isolate a single RF signal [32] and estimate its Doppler shift [33]. DD are calculated for all combinations of RF sensors and the sensor pair corresponding to the maximum DD is used to associate the RF sensors with the and multi-spectral tracker output. To associate the multi-spectral image tracker with the RF sensors, the absolute difference and root mean square difference (RMSD) is calculated and the sensor pair with the minimum metric is selected as the matching target. We compare the association rate of the sensors to the correct result to evaluate performance.

This algorithm was developed and evaluated using synthetically generated datasets. RF sensor measurements were simulated using the known ground truth radial velocity of the emanating target to generate Doppler shifts and varying levels of random measurement uncertainty were added. The imaging sensor is multi-spectral and includes visible, near-infrared (NIR), mid-wave infrared (MWIR), and long-wave infrared (LWIR) which were simulated using the Digital Imaging and Remote Sensing Image Generation (DIRSIG) software [34].

We present results on associating the RF sensors with the corresponding target from the multi-spectral video to localize and track a specific moving target with a cell phone. Using the absolute DD, the algorithm has a high rate of correctly associating the RF emanation with the multi-spectral target at low measurement uncertainty for targets that have motion patterns that are not similar to other targets. The RMSD improves the association rate particularly for low uncertainty cases, but maintains good performance through significant uncertainty levels. The confidence of identifying the correct multi-spectral target from background targets is high for low measurement uncertainty and remains high for over half of the targets through all uncertainty levels.

The remainder of the paper is organized as follows. Section 2 discusses the extraction of DD from RF sensors measuring a specific cellular emanation. Section 3 details the multi-spectral video tracker and the calculation of the theoretical DD. Section 4 discusses the metrics for associating the cellular emanation measured using the RF sensors with the output of the multi-spectral video tracking to localize a specific moving target. Experimental results for matching the cellular emanation as measured from the RF sensors with the video targets is discussed in Section 5. In Section 6 conclusions are presented.

## 2. Cell Phone Emanations

In this section we present background on sensing a specific cellular emanations from multiple RF receiver.

We start by reviewing the Doppler shift that occurs in a cellular emanation due to radial motion of the transmitter. A transmitting target has a position $\left(x_{k}^{t},y_{k}^{t},z_{k}^{t}\right)$ at time instance *k*. RF receivers are located at positions $\left(x_{k}^{\ell },y_{k}^{\ell },z_{k}^{\ell }\right)$, where *ℓ* is the receiver label. The range $R_{k}^{\ell }\left(\Delta x,\Delta y,\Delta z\right)$ between the transmitting target and a receiver *ℓ* is given

(1) $$R_{k}^{\ell }\left(\Delta x,\Delta y,\Delta z\right)=\sqrt{{\left(x_{k}^{\ell }-x_{k}^{t}\right)}^{2}+{\left(y_{k}^{\ell }-y_{k}^{t}\right)}^{2}+{\left(z_{k}^{\ell }-z_{k}^{t}\right)}^{2}}.$$

**Figure 5:**
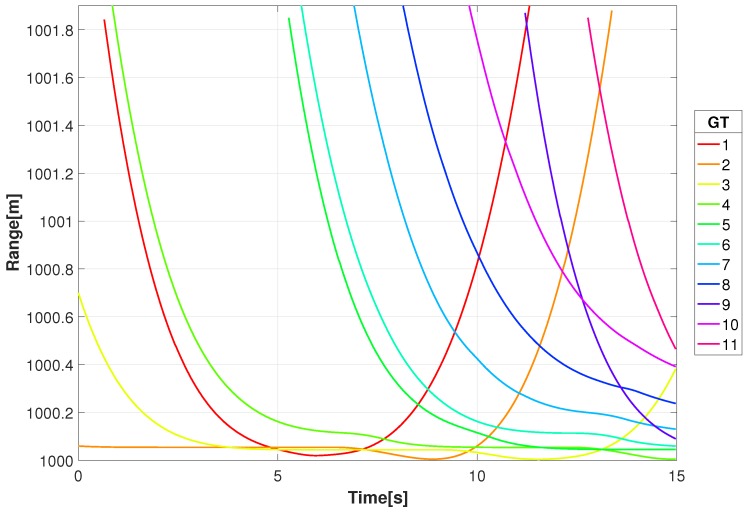
Range of the moving ground targets from the video sensor for the given video sequence.

Shown in Figure 5 are the ranges between the multi-spectral sensor $\left(x_{k}^{\ell }=0,y_{k}^{\ell }=0,z_{k}^{\ell }=1000\right)$ and the ground target (GT) vehicles as a function of time for the interval covered by the DIRSIG simulation. For the geometry and pattern flow of this scenario the targets enter at the edge of the scene, move towards the intersection at the center, and proceed to move towards the edge of the scene.

**Figure 6:**
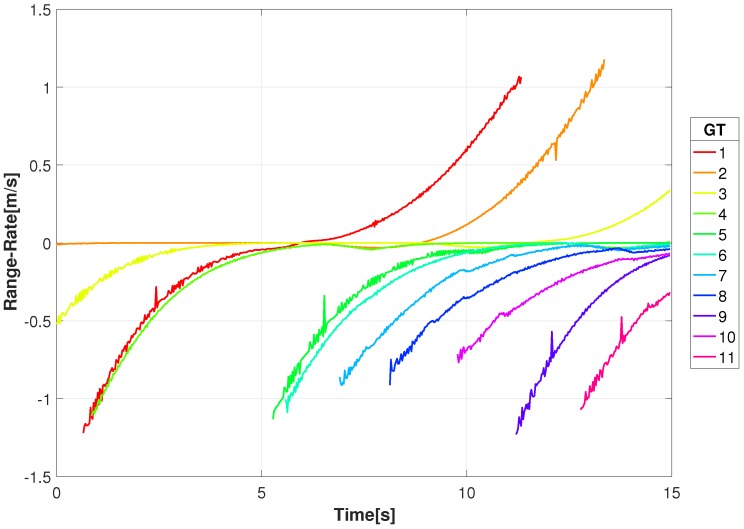
Range-rate of the moving ground targets from the video sensor for the given video sequence.

The derivative of the range with respect to time produces the range-rate *v*, also known as the radial velocity, and given by

(2) $$\begin{array}{c} v_{k}^{\ell }\left(\Delta x,\Delta y,\Delta z\right)=\frac{\partial }{\partial t}R_{k}^{\ell }\left(\Delta x,\Delta y,\Delta z\right)=\frac{\partial }{\partial t}\left(R_{k2}^{\ell }-R_{k1}^{\ell }\right)\\ \;=\frac{1}{T}\left(\sqrt{{\left(x_{k2}^{\ell }-x_{k2}^{t}\right)}^{2}+{\left(y_{k2}^{\ell }-y_{k2}^{t}\right)}^{2}+{\left(z_{k2}^{\ell }-z_{k2}^{t}\right)}^{2}}-\right.\\ \;\left.\sqrt{{\left(x_{k1}^{\ell }-x_{k1}^{t}\right)}^{2}+{\left(y_{k1}^{\ell }-y_{k1}^{t}\right)}^{2}+{\left(z_{k1}^{\ell }-z_{k1}^{t}\right)}^{2}}\right), \end{array}$$

where *T* is the sampling period between range measurements. Figure 6 is the range-rate for the GT in the scenario presented here. A negative range-rate indicates that the receiver and target are getting closer in range, and conversely, a positive range-rate indicates that the pair are moving away from one another. With the sensor altitude *z* being large compared to the (x,y) displacement and the motion being confined to the *x*−*y* plane, the large ranges and low ground velocities result in low range-rates. The similarity of the traffic patterns is due to the geometry of the scene and the traffic scenario. This introduces difficulty in distinguishing between targets since the Doppler shifts will be small in general, and when vehicles stop, for example at traffic lights, it will disappear. By using spatially separated receivers we create diversity in the Doppler shifts and create the opportunity to use the highest Doppler shifts which will least likely be associated with non-moving targets.

**Figure 7:**
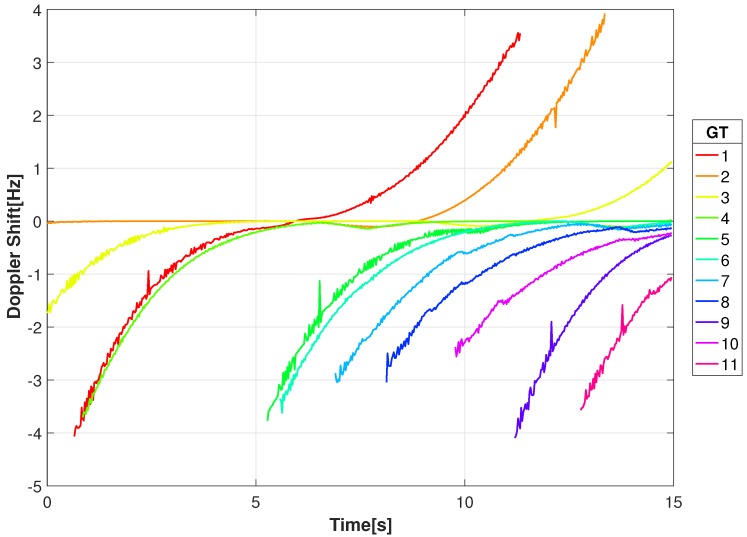
Doppler Shift at 1 GHz carrier frequency for the moving ground targets as measured from location of the video sensor for the given video sequence.

We now examine the effect of the radial velocity on the frequency $f_{c}$ of a carrier signal for a cellular emanation. A transmitter with a radial velocity in the direction of a stationary receiver results in a shift of frequency that makes it larger, whereas radial velocity in the opposing direction of the receiver results in a frequency shift that makes it smaller. This is a well known concept called the Doppler effect. The shift in frequency $\Delta f=f_{D}-f_{c}$ is given by

(3) $$\Delta f=f_{c}\left(1+\frac{v_{k}}{c}\right),$$

where *c* is the speed of light. Shown in Figure 7 are the Doppler shifts corresponding to the radial velocities from Figure 6 for a carrier frequency of 1 GHz. For the 1 GHz carrier the Doppler shifts range between −4 and 4 Hz.

Similar motion profiles resulting from using a single sensor make it difficult to distinguish between targets. The use of a constellation of spatially separated receivers produces variation in the Doppler signature for the targets. For the purpose of this study eight RF receivers were placed on the edge of the imaging sensors field of view and one in the center; locations are shown in Figure 2. The spatial distribution of the sensors is such that some will be in the direction of travel and range will be decreasing, resulting in a negative range-rate and a negative Doppler shift. The other sensors will be opposite the direction of travel and the range will increase, resulting in a positive range-rate and positive shift in frequency.

**Figure 8:**
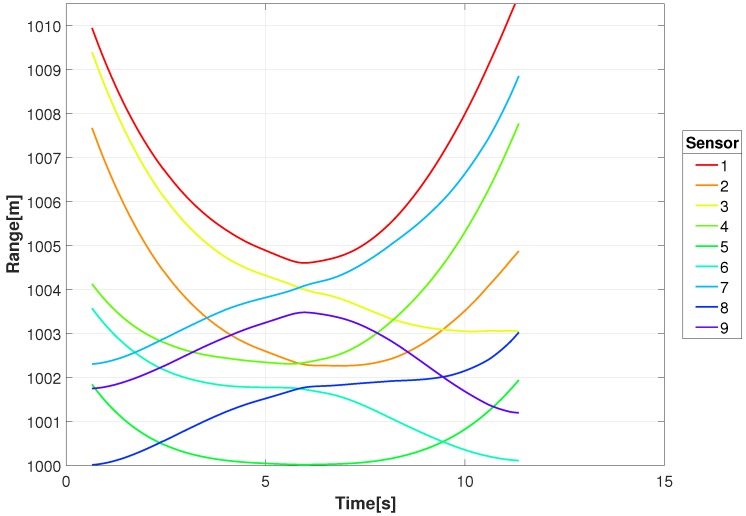
Example ranges between a transmitting target (GT#1) and the locations of the RF sensor constellation.

**Figure 9:**
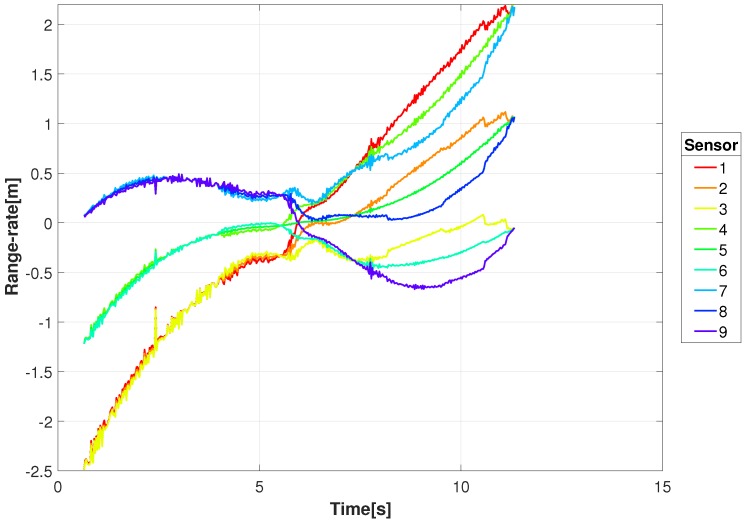
Example range-rate between a transmitting target (GT #1) and the locations of the RF sensor constellation.

**Figure 10:**
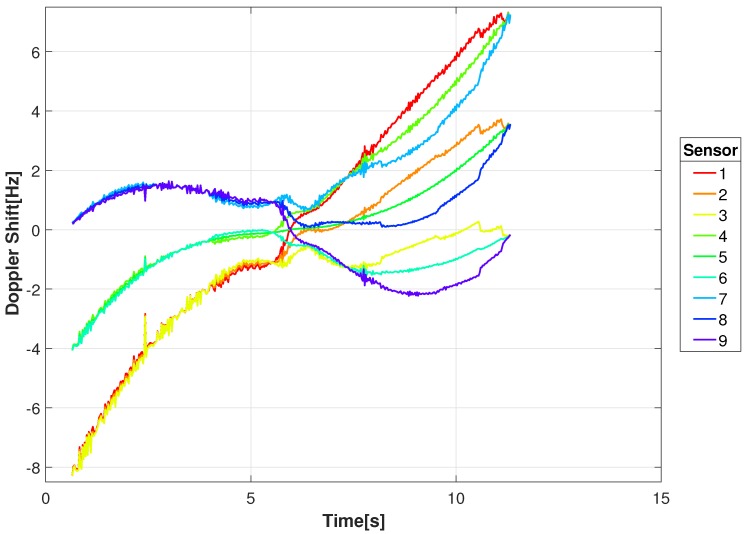
Example Doppler shifts for 1 GHz carrier frequency between a transmitting target (GT #1) and the locations of the RF sensor constellation.

Figure 8 shows the range between an example transmitting target (GT #1) and the constellation sensor locations in Figure 2. As indicated, some sensors are decreasing in range while others are increasing. This is better illustrated in Figure 9 and Figure 10 where the range-rate and Doppler shift for a 1 GHz carrier are shown, respectively. At the beginning of movement for this example, the Doppler shift for the 1 GHz carrier has a shift near −8 Hz for three sensors and a shift near 0 Hz for three sensors, giving a difference of 8 Hz. That difference decreases as the target approaches the intersection with decreasing speed and eventually comes to a stop around 6 s, resulting in nearly 0 Hz difference in Doppler shift.

The DD $\rho_{k}$ is defined as the difference in Doppler shift between receivers ℓ1 and ℓ2 and is given by

(4) $$\rho_{k}=\Delta f_{k}^{\ell 1}-\Delta f_{k}^{\ell 2}.$$

**Figure 11:**
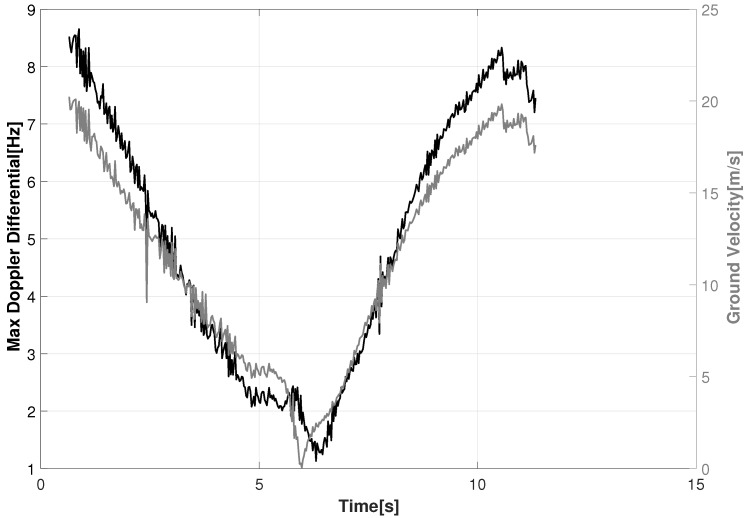
Maximum Doppler differential for GT #1 using all RF sensor combinations (left *Y*-axis) against its corresponding ground speed (right *Y*-axis).

The DD varies between RF sensor pairs based on their geometry and the radial velocity of the moving target. For example, one target may be stationary and have no DD, whereas another target may be moving radially with respect to the sensors which results in a near zero DD (but may be non-zero to other sensor pairs). The maximum DD selects the sensors that are positioned orthogonal to the targets motion. An example of the maximum DD is shown in Figure 11 for GT #1 with the corresponding ground speed. The target starts out with its highest DD when it first enters the scene and decelerates as it moves towards the intersection at the center of the scene and reaches a DD of 1 Hz. After reaching the intersection the target increases in speed and the DD increases to 8.3 Hz.

## 3. Multi-Spectral Video Tracker

**Figure 12:**
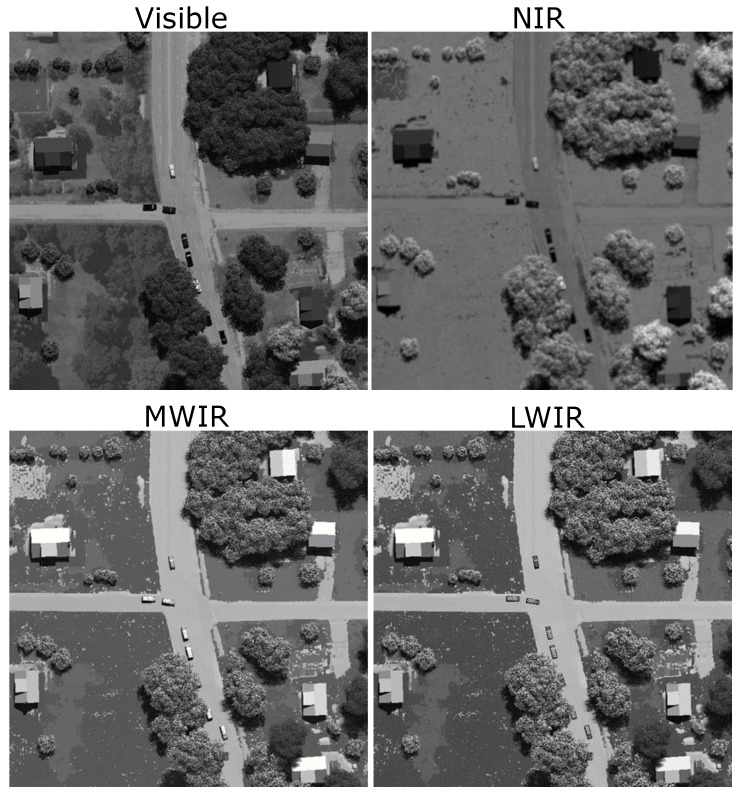
Example multi-spectral images frames from the synthetic DIRSIG data set.

We present an overview of the algorithm to fuse multi-spectral video data to detect and track moving targets in a cluttered urban environment [31]. The algorithm was developed and tested using a sythetically generated dataset produced using the DIRSIG toolset in visible, NIR, MWIR, and LWIR spectral bands [34]. Figure 12 shows an example frame of each spectral band with a frame size of 2000 × 2000-pixels and a ground sample distance of 0.0635 m, resulting in a field of view covering 137 × 137 m^2^. By visual inspection of the frames in Figure 12, the appearance of target vehicles varies between the spectral bands, providing different intensity information. The vehicle motion in the video sequence was simulated as a common traffic pattern using the open source tool Simulation of Urban MObilitiy (SUMO) to provide realistic traffic maneuvers [35].

The intensity of a pixel fluctuates due to noise, changes in illumination, and movement from both clutter and target objects. This does not allow a single value to represent the time history of the intensity of a single pixel for a given sequence of video frames. To compensate for these changes, background modeling techniques are used to describe the probability distribution of the pixels intensity by empirically deriving and updating the parameters from the video sequence. The Gaussian mixture model (GMM) has been successfully demonstrated to model the fluctuations in pixel intensities in outdoor scenes for detection of pedestrians [17,18] and vehicles [19,36].

To deal with fluctuating pixel intensities in our video sequence, in each spectral band we use a GMM that adapts to the evolving scene by modeling the time history of intensity of each pixel to determine the foreground pixels. The GMM extracts the foreground pixels by modeling the background distribution of intensity at each pixel by a number of Gaussian distributions, and a pixel not fitting these distributions is classified as a foreground pixel. A fused foreground map is created by weighting and summing foreground pixels from all spectral bands. A threshold is applied to the fused foreground map to remove low weighted pixels and an image closing operation is performed to create pixel groups labeled as targets.

Targets are associated between frames by relating historical track information constructed from prior tracked frames with position estimates in the current frame. Relating track history to current positional data is trivial in the scenarios where targets stay separated and no occlusions exist. However, in actual practice and in this data set, targets can be merged and appear as a single target, or occluded by trees, etc., making distinguishing between targets and maintaining the correct association of tracker data and target difficult. Features using the scale-invariant feature transforms (SIFT) were selected for identification of targets due to their robustness with respect to changes in rotation and scale, and their invariance to change in camera viewpoints and illumination changes [37,38]. SIFT features are composed of a keypoint that has a sub-pixel location estimate and the gradient orientation of the feature, along with a descriptor that is calculated based on histogram of the local pixel texture.

**Figure 13:**
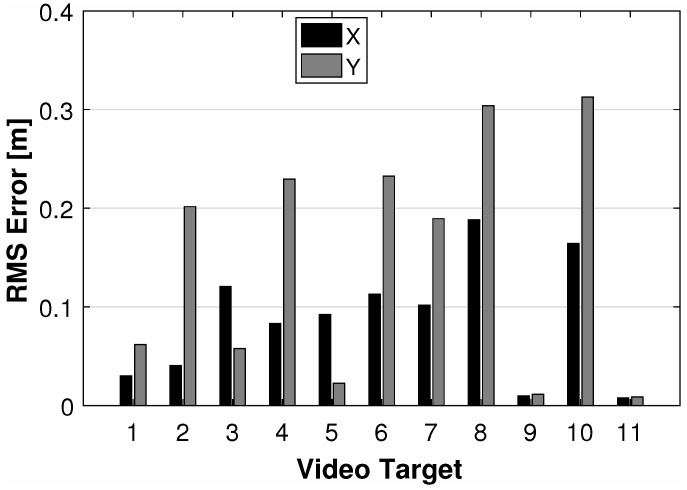
RMSE of the centroid estimate using the multi-spectral video tracker for the ground-truthed video targets.

Results from the multi-spectral imaging system are a collection of tracked targets that have correlated (x,y) centroid measurements in the imaging plane. The root-mean-square error (RMSE) between the true and estimated centroid locations for the video tracked objects are shown in Figure 13. Video targets 8 and 10 have the largest RMSE where the *y*-error is over 0.3 meters and the *x*-error is over 0.15 m. The other targets have a considerably lower RMSE in both *x* and *y*.

Theoretical DD for the output targets of the multi-spectral tracker are calculated using Equations (1)–(4) where target positions $x_{k}^{t}$ and $y_{k}^{t}$ are ground coordinates from the measured video frames and $z_{k}^{\ell }$ is the ground height which is 0 meters for this scenario. Sensor coordinates $\left(x_{k}^{\ell },y_{k}^{\ell },z_{k}^{\ell }\right)$ are the locations of the RF sensors.

## 4. Data Association

**Figure 14:**
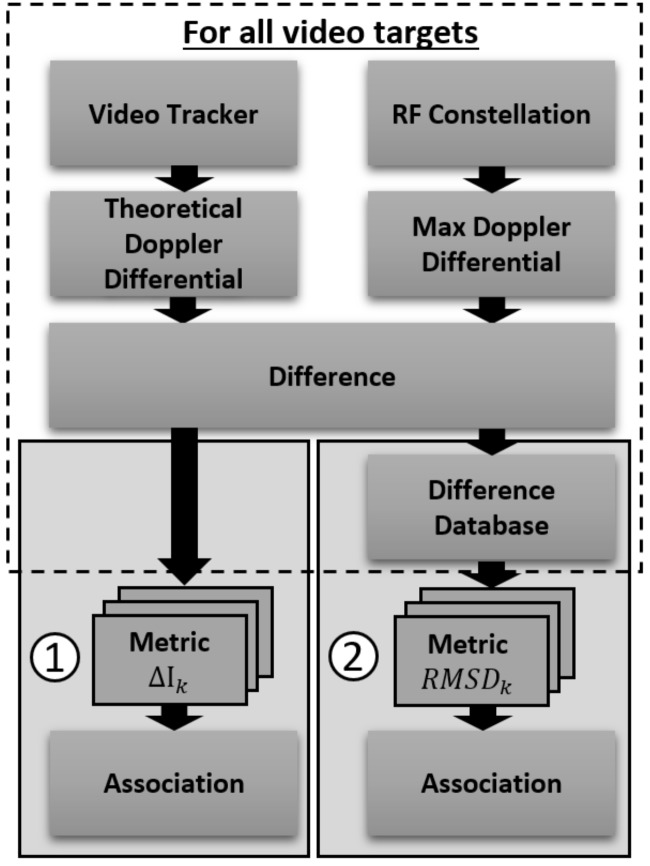
The process of associating the DD at a given frame measured from a constellation of RF sensors with the output of a multi-spectral video tracker using two statistical metrics; (1) absolute difference and (2) root-mean-square difference.

The data fusion process associates a specific cellular emanation measured by the constellation of RF sensors with the corresponding target in the output of the multi-spectral video tracker. A flowchart of the process to associate the two sensors at frame *k* is shown in Figure 14. The multi-spectral video tracker produces estimates of the radial velocity computed from two dimensional tracker output which is used to calculate the theoretical DD between the tracked target and locations of the RF sensors. The RF constellation produces measurements of the DD from all combinations of RF receivers and the pair that maximizes the magnitude of the DD is selected. The difference in DD between the RF sensors and the multi-spectral video sensor is calculated for all video tracked targets. We propose two metrics to associate the max DD from the RF sensor measurements with the theoretical DD from multi-spectral video tracker output; (1) the absolute difference $\Delta I_{k}$ and (2) the root-mean-square difference $RMSD_{k}$. In the case of metric (2) the difference in DD is stored in a database for the time averaged calculation. The absolute difference $\Delta I_{k}$ at frame *k* between the DD of RF sensors $\rho_{k}^{RF}$ and the video tracker output $\rho_{k}^{vid}$ is given

(5) $$\Delta I_{k}=\left|\rho_{k}^{RF}-\rho_{k}^{vid}\right|.$$

This absolute difference is particularly well suited for systems that do not have adequate samples to produce a statistical average due to sparse measurements by either the RF or video sensors. The root-mean-square difference $RMSD_{k}$ is a time averaged difference in DD between the RF sensors and the video sensor given by

(6) $$RMSD_{k}=\sqrt{\frac{\sum_{n=1}^{N}{\left(\rho_{n}^{RF}-\rho_{n}^{vid}\right)}^{2}}{N}},$$

where *N* is the number of averaged measurements. The $RMSD_{k}$ metric lowers false associations that are attributed to sporadic measurement error which may occur using $\Delta I_{k}$.

## 5. Experiment

We present the performance of the fusion algorithm for associating a specific cellular emanation detected from a constellation of RF receivers with the corresponding target from the multi-spectral video tracker. An association is classified as correct when the cellular emanation from the RF sensors is correctly matched with the corresponding video target. Performance of the algorithm was evaluated using Doppler measurements from the RF receivers with added measurement uncertainty and (x,y) centroid locations from a multi-spectral video tracker. Doppler shifts for a cellular frequency were generated from the ground truth radial velocity with varying RMS levels of white Gaussian noise added to the Doppler shifts to model measurement uncertainty. Centroid location information was extracted from a multi-spectral video set that was developed with the DIRSIG software tool [34] with the detection and tracking algorithm presented in [31]. The results are the correct association rates over the video sequence for 100 Monte Carlo simulations.

**Figure 15:**
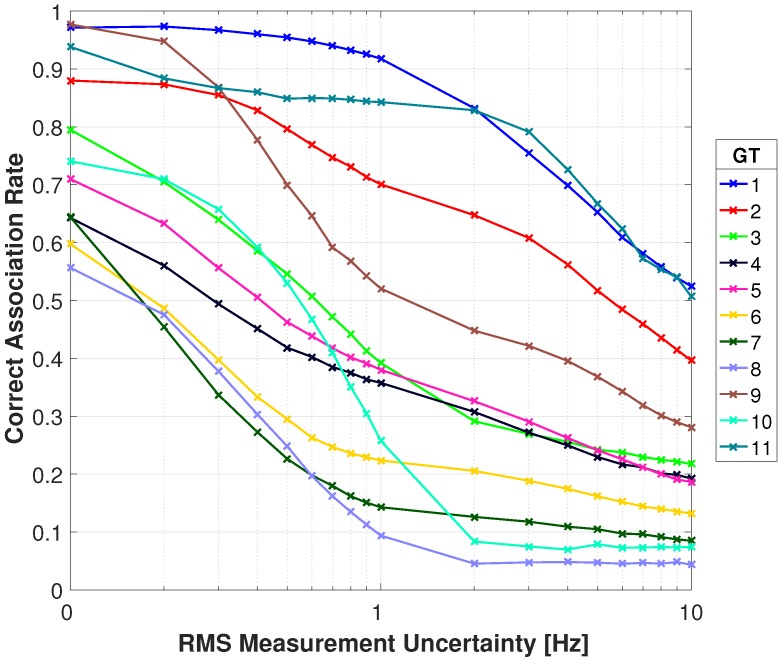
Correct association rate for matching specific cellular emanation measured by the constellation of RF receivers with the corresponding target from the multi-spectral video tracker using the absolute DD.

**Figure 16:**
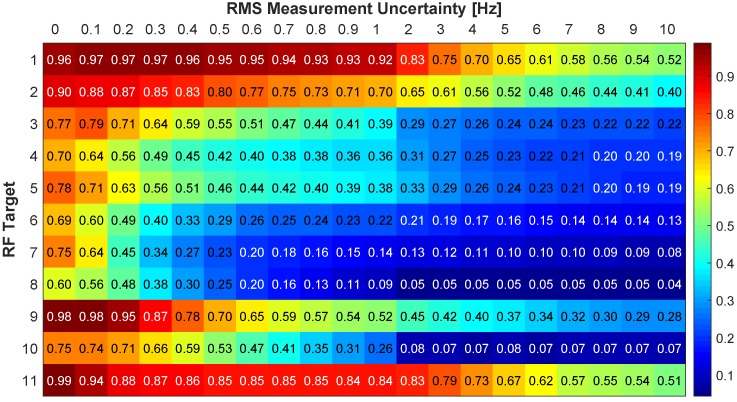
Correct association rate for matching specific cellular emanation measured by the constellation of RF receivers with the corresponding target from the multi-spectral video tracker using the absolute DD.

Figure 15 and Figure 16 show the association rates using the absolute difference metric. With no uncertainty in the Doppler measurement targets 1, 9, and 11 have association rates above 0.96. Target 1 maintains a rate of 0.92 through 1 Hz of RMS uncertainty and has a gradual decrease to 0.52 at 10 Hz RMS uncertainty. The association rate for target 11 drops to 0.84 with 1 Hz RMS uncertainty, and has a slow decrease through 10 Hz which has a rate of 0.51. Target 2 has a steeper decrease in rate and drops to 0.7 with 1 Hz RMS uncertainty and decreases to 0.40 with 10 Hz. Remaining targets have an association rate greater than 0.60 with no uncertainty. Targets 4 and 6–8 have the poorest performance as they drop below 0.5 with 0.2 Hz RMS uncertainty. Overall the results with no Doppler uncertainty are promising, but with added uncertainty there is a significant decrease in performance.

**Figure 17:**
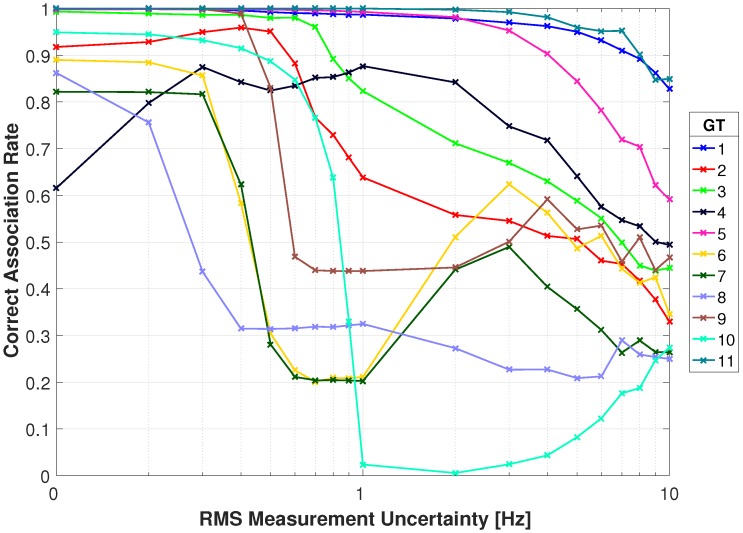
Correct association rate for matching specific cellular emanation measured by the constellation of RF receivers with the corresponding target from the multi-spectral video tracker using the RMSD DD.

**Figure 18:**
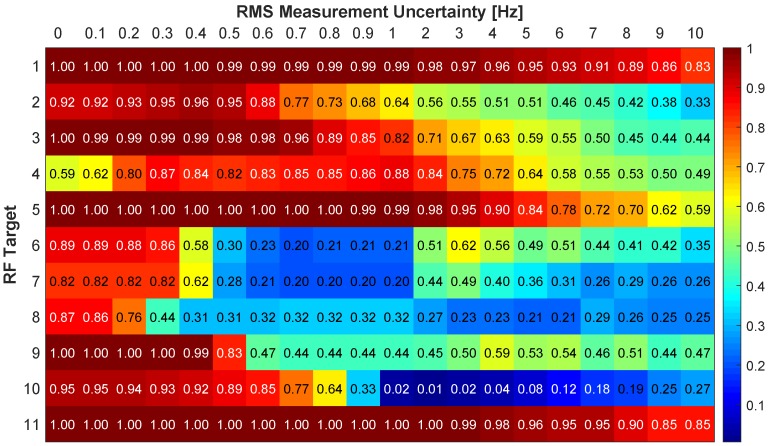
Correct association rate for matching specific cellular emanation measured by the constellation of RF receivers with the corresponding target from the multi-spectral video tracker using the RMSD DD.

Figure 17 and Figure 18 show the association rates using the RMSD. With no added Doppler uncertainty all targets start off with an association rate above 0.82 excluding target 4. Targets 1, 3, 5, 9, and 11 have perfect association rates with 7 of the 11 targets being 0.92 or greater. Targets 1 and 11 have a gradual decrease in association rate and maintain a rate greater than 0.83 through 10 Hz of RMS uncertainty. Target 5 has an association rate greater than 0.9 through 4 Hz and gradually decreases to 0.59 at 10 Hz. Target 2 has a rate above 0.92 through 0.5 Hz of RMS uncertainty and gradually decreases to below 0.51 at 5 Hz. Targets 6 and 7 drop below 0.62 at 0.4 Hz of RMS uncertainty due to similar Doppler signatures. Target 10 has a rate above 0.77 through 0.7 Hz RMS uncertainty but begins to decrease significantly. This target has poor performance in the video tracker and a similar DD signature as target 2. Target 8 has the most immediate decrease in performance by dropping below 0.44 at 0.3 Hz due to a similar Doppler signature as target 4. There is an improvement in performance for some targets as uncertainty is increased, particularly target 4 when increasing from no uncertainty through 0.3 Hz. This is attributed to similar Doppler signatures for a particular sensor configuration. With added uncertainty a different sensor pair produces a higher DD, and that sensor pair proves to have better performance with the multi-spectral video tracker. For example, with no RMS uncertainty target 4 has multiple sensor pairs with nearly identical DD, with a slightly higher DD for Sensor 1 and Sensor 9. As RMS uncertainty is added, this optimal sensor pair begins to switch between different pairs (i.e. Sensor 1 & Sensor 9, Sensor 4 & Sensors 6, Sensor 6 & Sensor 7). These new sensor pairs produce DD for target 2 that are not similar to target 4, reducing the incorrect associations. Overall, these results are improved in comparison to the absolute difference and provide robustness to measurement uncertainty.

The confidence ratio CR is a performance measure of confidence for correctly identifying the true video target from the most probable incorrect target after all frames measurements have been made. It is defined as the normalized difference ratio between the number of correct associations Corr and the number of associations for the highest detected incorrect target Bkg and given by

(7) $$CR=\frac{Corr-Bkg}{Corr+Bkg}.$$

A positive CR detects the correct target more than an incorrect target, where a maximum value of 1.0 indicates that the correct association was made for all frames. A negative CR detects an incorrect target more than the correct target, where a value of −1.0 indicates that the incorrect association was made for all frames. This metric gives us a value on how likely we are to differentiate the true target from the most probable incorrect target.

**Figure 19:**
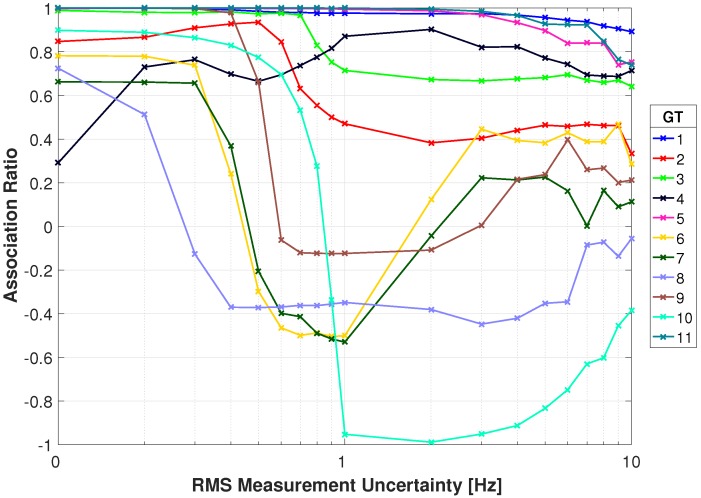
Confidence ratio for matching specific cellular emanation measured by the constellation of RF receivers with the corresponding target from the multi-spectral video tracker using the RMSD DD.

**Figure 20:**
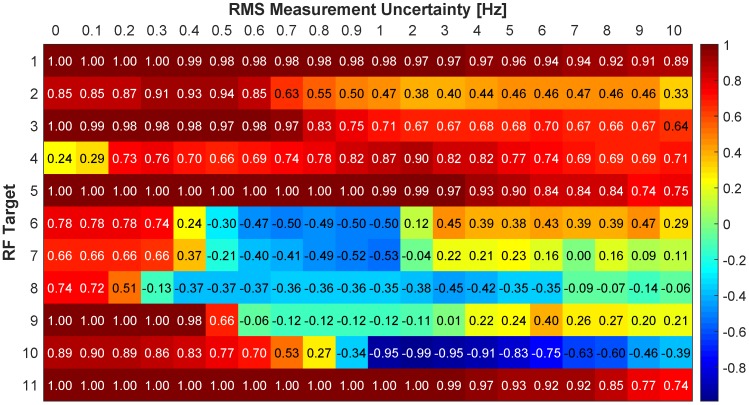
Confidence ratio for matching specific cellular emanation measured by the constellation of RF receivers with the corresponding target from the multi-spectral video tracker using the RMSD DD.

Figure 19 and Figure 20 show the confidence ratios. Targets 1, 3, 5, 9, and 11 have an CR of 1.0 with no Doppler uncertainty. Targets 1, 5, and 11 maintain a CR greater than 0.75 through 10 Hz RMS uncertainty while target 3 maintains a ratio greater than 0.6. The CR for target 6 and 7 becomes negative at 0.5 Hz of RMS uncertainty, but becomes positive again at 3 Hz. The increase in CR is attributed to Bkg becoming distributed between multiple incorrect targets, resulting in a lower Bkg for the single target. Due to the similarity with another target at the beginning of the time interval, the CR for target 4 begins low but after 0.1 Hz of RMS uncertainty it increases above 0.66 for all levels of uncertainty. Target 10 has the lowest CR at 1 Hz RMS uncertainty and remains low for all levels. Target 10 has the highest RMSE in the video tracker and has a similar DD signature to target 2. For targets 1–5 and 11 the confidence is positive for all uncertainty levels, indicating we can successfully associate the RF emanations with the corresponding target from the multi-spectral video tracker through all uncertainty levels.

## 6. Conclusions

In this paper, we proposed an algorithm to localize and track a specific target by fusing data from a constellation of RF receivers measuring Doppler shifts from a specific cell phone with the output from a multi-spectral video tracker. This work is unique from other fusion literature in that it fuses a new set of sensors to localize and track a specific moving target. The constellation of RF sensors measure the Doppler shift from a cellular emanation in a specific moving vehicle and the DD between all sensor pairs is calculated. The multi-spectral video tracker uses a GMM to detect foreground objects and SIFT features to track them, and produces (x,y) centroid locations of detected vehicles. The specific target is localized by associating the DD from the RF sensors with the theoretical DD calculated from the multi-spectral video tracker by comparing the DD using two metrics; the absolute and RMSD DD. Using synthetically generated data, results demonstrate we successfully associate cellular emanations with their corresponding target in a multi-spectral video tracker, but measurement uncertainty and motions patterns affect the correct association rate. The confidence of identifying the correct multi-spectral target from the most probable background target is high for low measurement uncertainty and remains high for over half of the targets through all uncertainty levels.

## References

[1] Adams A., Tummala M., McEachen J., Scrofani J. Source localization and tracking in a Cognitive Radio environment consisting of frequency and spatial mobility. Proceedings of the 7th International Conference on Signal Processing and Communication Systems (ICSPCS).

[2] Hall D.L., Llinas J. (1997). An introduction to multisensor data fusion. Proc. IEEE.

[3] Atrey P.K., Hossain M.A., El Saddik A., Kankanhalli M.S. (2010). Multimodal fusion for multimedia analysis: A survey. Multimed. Syst..

[4] Tan D.K., Sun H., Lu Y., Lesturgie M., Chan H.L. (2005). Passive radar using global system for mobile communication signal: theory, implementation and measurements. IEE Proc. Radar Sonar Navig..

[5] Griffiths H., Baker C. (2005). Passive coherent location radar systems. Part 1: performance prediction. IEE Proc. Radar Sonar Navig..

[6] Lee B., Chan Y., Chan F., Du H.J., Dilkes F.A. Doppler frequency geolocation of uncooperative radars. Proceedings of the MILCOM 2007-IEEE Military Communications Conference.

[7] Vasuhi S., Vaidehi V. (2016). Target tracking using interactive multiple model for wireless sensor network. Inf. Fusion.

[8] Zhang Y.D., Himed B. Moving target parameter estimation and SFN ghost rejection in multistatic passive radar. Proceedings of the 2013 IEEE Radar Conference (RadarCon13).

[9] Kim W.S. (2015). The Fourth Amendment Implication on the Real-Time Tracking of Cell Phones through the Use of Stingrays. Fordham Intell. Prop. Med. Ent. Law J..

[10] Pell S.K., Soghoian C. (2014). Your secret stingray’s no secret anymore: The vanishing government monopoly over cell phone surveillance and its impact on national security and consumer privacy. Harv. J. Law Technol..

[11] Dai C., Zheng Y., Li X. (2007). Pedestrian detection and tracking in infrared imagery using shape and appearance. Comput. Vision Image Underst..

[12] Li J., Gong W., Li W., Liu X. (2010). Robust pedestrian detection in thermal infrared imagery using the wavelet transform. Infrared Phys. Technol..

[13] Song X., Shao X., Zhang Q., Shibasaki R., Zhao H., Zha H. (2013). A novel dynamic model for multiple pedestrians tracking in extremely crowded scenarios. Inf. Fusion.

[14] Heintz F., Rudol P., Doherty P. From images to traffic behavior-a uav tracking and monitoring application. Proceedings of the 10th International Conference on Information Fusion.

[15] Meng L., Kerekes J.P. (2012). Object tracking using high resolution satellite imagery. IEEE J. Sel. Top. Appl. Earth Obs. Remote Sens..

[16] Rastegar S., Babaeian A., Bandarabadi M., Toopchi Y. Airplane detection and tracking using wavelet features and SVM classifier. Proceedings of the 41st Southeastern Symposium on System Theory.

[17] Stauffer C., Grimson W.E.L. Adaptive background mixture models for real-time tracking. Proceedings of the IEEE Computer Society Conference on Computer Vision and Pattern Recognition.

[18] KaewTraKulPong P., Bowden R. (2002). An improved adaptive background mixture model for real-time tracking with shadow detection. Video-Based Surveillance Systems.

[19] Zivkovic Z. Improved adaptive Gaussian mixture model for background subtraction. Proceedings of the 17th International Conference on Pattern Recognition.

[20] Maini R., Aggarwal H. (2009). Study and comparison of various image edge detection techniques. Int. J. Image Process. (IJIP).

[21] Tuytelaars T., Mikolajczyk K. (2008). Local invariant feature detectors: A survey. Found. Trends Comput. Graphics Vision.

[22] Noulas A., Englebienne G., Krose B.J. (2012). Multimodal speaker diarization. IEEE Trans. Pattern Anal. Mach. Intell..

[23] Kılıiç V., Zhong X., Barnard M., Wang W., Kittler J. Audio-visual tracking of a variable number of speakers with a random finite set approach. Proceedings of the 17th International Conference on Information Fusion (FUSION).

[24] D’Arca E., Robertson N.M., Hopgood J. Person tracking via audio and video fusion. Proceedings of the 9th IET Data Fusion Target Tracking Conference (DF TT 2012): Algorithms and Applications.

[25] Chin T., Xiong K., Blasch E. Nonlinear target tracking for threat detection using RSSI and optical fusion. Proceedings of the 18th International Conference onInformation Fusion (Fusion).

[26] Weinstein E. (1982). Measurement of the differential Doppler shift. IEEE Trans. Acoust. Speech Signal Process..

[27] Otnes R.K. (1989). Frequency difference of arrival accuracy. IEEE Trans. Acoust. Speech Signal Process..

[28] Tenenbaum S., Stouch D., McGraw K., Fichtl T. Multi-objective optimization to support mission planning for constellations of unmanned aerial systems. Proceedings of the SPIE Defense and Security Symposium.

[29] Danoy G., Brust M.R., Bouvry P. Connectivity stability in autonomous multi-level UAV swarms for wide area monitoring. Proceedings of the 5th ACM Symposium on Development and Analysis of Intelligent Vehicular Networks and Applications.

[30] Stouch D.W., Zeidman E., Callahan W., McGraw K. Dynamic replanning on demand of UAS constellations performing ISR missions. Proceedings of the SPIE Defense and Security Symposium.

[31] Demars C.D., Roggemann M.C., Havens T.C. (2015). Multispectral detection and tracking of multiple moving targets in cluttered urban environments. Opt. Eng..

[32] Tse D., Viswanath P. (2005). Fundamentals of Wireless Communication.

[33] Kay S. (2013). A computationally efficient nonlinear least squares method using random basis functions. IEEE Signal Process. Lett..

[34] Sanders J.S., Brown S.D. (2000). Utilization of DIRSIG in support of real-time infrared scene generation. Proc. SPIE.

[35] Behrisch M., Bieker L., Erdmann J., Krajzewicz D. Sumo-simulation of urban mobility-an overview. Proceedings of the Third International Conference on Advances in System Simulation.

[36] Chen Z., Ellis T. Self-adaptive Gaussian mixture model for urban traffic monitoring system. Proceedings of the IEEE International Conference on Computer Vision Workshops (ICCV Workshops).

[37] Lowe D.G. (2004). Distinctive image features from scale-invariant keypoints. Int. J. Comput. Vision.

[38] Vedaldi A., Fulkerson B. VLFeat: An Open and Portable Library of Computer Vision Algorithms. http://www.vlfeat.org.

